# Supplementary material for: Protective immunity against malaria by a nanoparticle CIS43-based junctional vaccine alone or in combination with R21
Source: NPJ Vaccines. 2026 Apr 27;11:133. doi: 10.1038/s41541-026-01455-3 (PMC13324253; doi:10.1038/s41541-026-01455-3)
Supplement: Supplementary file 1 — Supplementary information [file 41541_2026_1455_MOESM1_ESM.pdf]

Supplementary Table 1. Immunogen sequences.

Construct name<br><br>(Nterm attachment-nanoparticle-number of repeats-Cterm attachment)	Amino acid sequence
**NPDP19-LuS-PADRE**<br>N-term attachment – NPDP19<br>Nanoparticle- LuS<br>Number of repeats – 1<br>C-term attachment – PADRE	mdskgssqkgsrlllllvvsnlllpqgvvgKQPADGNPDPNANPNVDPNgsgsaMQIYEGKLTA<br>EGLRFGIVASRFNHALVDRLVEGAIDAIVRHGGREEDITLVRVPGSWEIPVAAGE<br>LARKENISAVIAIGVLIRGATPHFDYIASEVSKGLADLSLELRKPITFGVITADTLEQ<br>AIERAGTKHGNKGWEAALSAIEMANLFKSLRGAKFVAAWTLKAAA
**2T-LuS-PADRE**<br>N-term attachment – NPDP19<br>Nanoparticle- LuS<br>Number of repeats – 2 in tandem<br>C-term attachment – PADRE	mdskgssqkgsrlllllvvsnlllpqgvvgKQPADGNPDPNANPNVDPNggsggKQPADGNPD<br>PNANPNVDPNgsgsaMQIYEGKLTAEGLRFGIVASRFNHALVDRLVEGAIDAIVRH<br>GGREEDITLVRVPGSWEIPVAAGELARKENISAVIAIGVLIRGATPHFDYIASEVS<br>KGLADLSLELRKPITFGVITADTLEQAIERAGTKHGNKGWEAALSAIEMANLFKSL<br>RGAKFVAAWTLKAAA
**PADRE-LuS-2T**<br>N-term attachment – PADRE<br>Nanoparticle- LuS<br>Number of repeats – 2 in tandem<br>C-term attachment – NPDP19	mdskgssqkgsrlllllvvsnlllpqgvvgAKFVAAWTLKAAASLVRgsgsaMQIYEGKLTAEGL<br>RFGIVASRFNHALVDRLVEGAIDAIVRHGGREEDITLVRVPGSWEIPVAAGELAR<br>KENISAVIAIGVLIRGATPHFDYIASEVSKGLADLSLELRKPITFGVITADTLEQAIE<br>RAGTKHGNKGWEAALSAIEMANLFKSLRGggsggKQPADGNPDPNANPNVDPN<br>ggsggKQPADGNPDPNANPNVDPN
**3T-LuS-PADRE**<br>N-term attachment – NPDP19<br>Nanoparticle- LuS<br>Number of repeats – 3 in tandem<br>C-term attachment – PADRE	mdskgssqkgsrlllllvvsnlllpqgvvgKQPADGNPDPNANPNVDPNggsggKQPADGNPD<br>PNANPNVDPNggsggKQPADGNPDPNANPNVDPNgsgsaMQIYEGKLTAEGLRF<br>GIVASRFNHALVDRLVEGAIDAIVRHGGREEDITLVRVPGSWEIPVAAGELARKE<br>NISAVIAIGVLIRGATPHFDYIASEVSKGLADLSLELRKPITFGVITADTLEQAIERA<br>GTKHGNKGWEAALSAIEMANLFKSLRGAKFVAAWTLKAAA
**PADRE-LuS-3T**<br>N-term attachment – PADRE<br>Nanoparticle- LuS<br>Number of repeats – 3 in tandem<br>C-term attachment – NPDP19	mdskgssqkgsrlllllvvsnlllpqgvvgAKFVAAWTLKAAASLVRgsgsaMQIYEGKLTAEGL<br>RFGIVASRFNHALVDRLVEGAIDAIVRHGGREEDITLVRVPGSWEIPVAAGELAR<br>KENISAVIAIGVLIRGATPHFDYIASEVSKGLADLSLELRKPITFGVITADTLEQAIE<br>RAGTKHGNKGWEAALSAIEMANLFKSLRGggsggKQPADGNPDPNANPNVDPN<br>ggsggKQPADGNPDPNANPNVDPNggsggKQPADGNPDPNANPNVDPN

* Red: signal sequence; blue: PADRE; orange: cleavage site; purple: linker

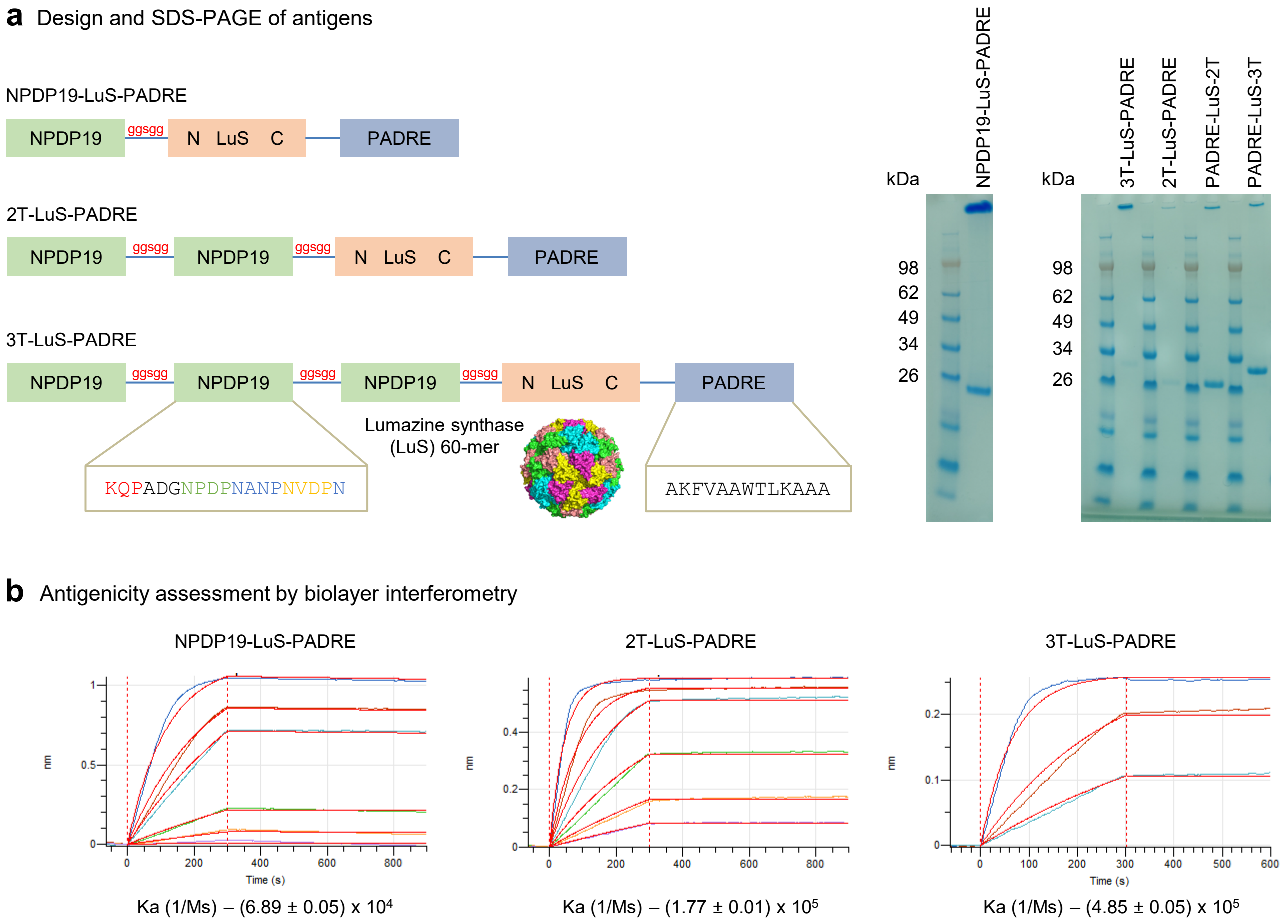

Supplementary Figure 1. Characterization of immunogens by SDS-PAGE and biolayer interferometry, related to Fig 1.
a Design schematic and SDS-PAGE of antigens. **b** Binding assessment of antigens to antibody D3 by biolayer interferometry.

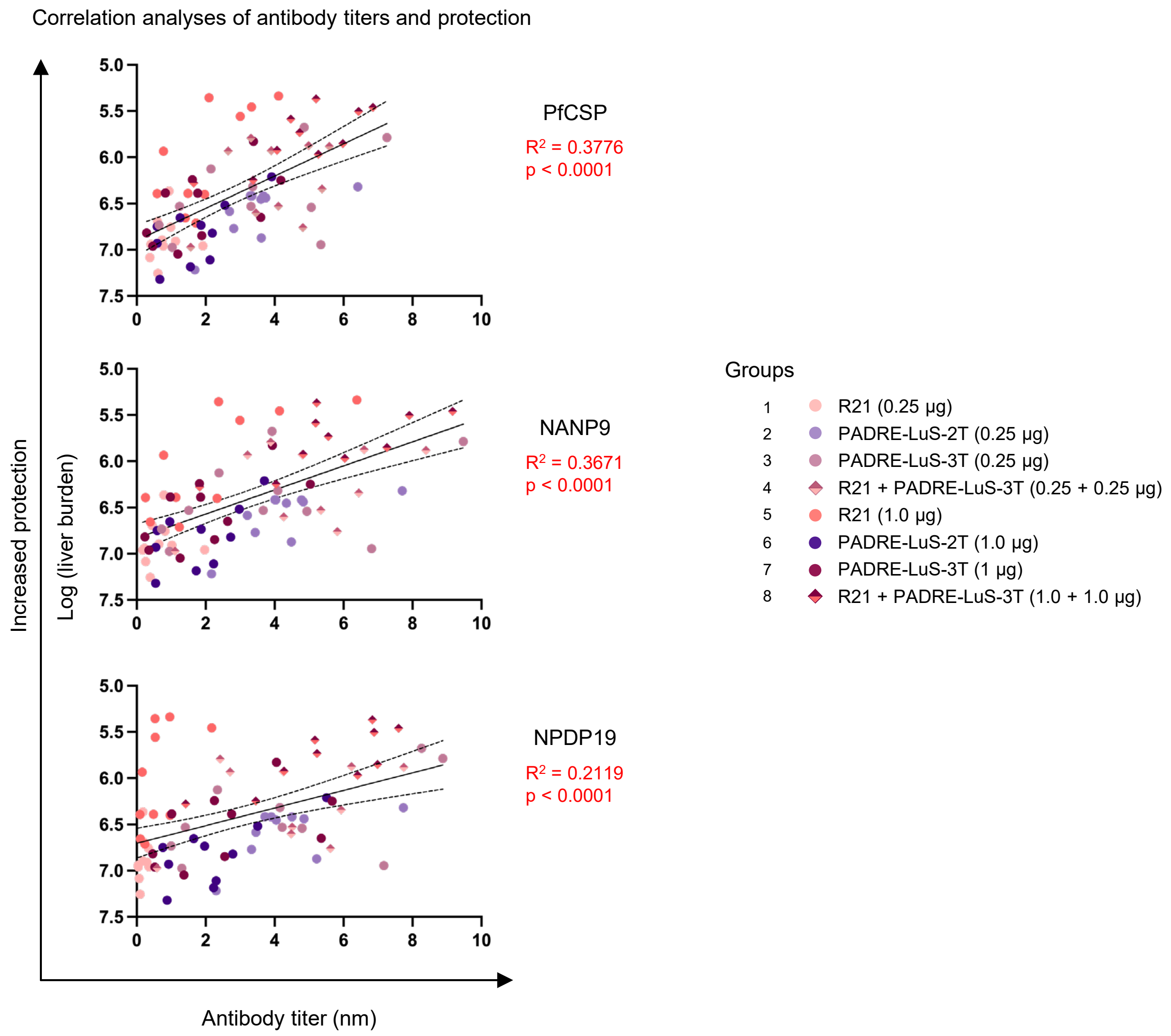

Supplementary Figure 2. Protective efficacy and antibody titers correlation, related to Fig 4.
Correlation analyses for week 6 antibody titers and liver burden (total flux) for three probes; PfCSP, NANP9 (major), and NPDP19 (junction). Simple linear regression analysis was performed in GraphPad Prism version 9.0.

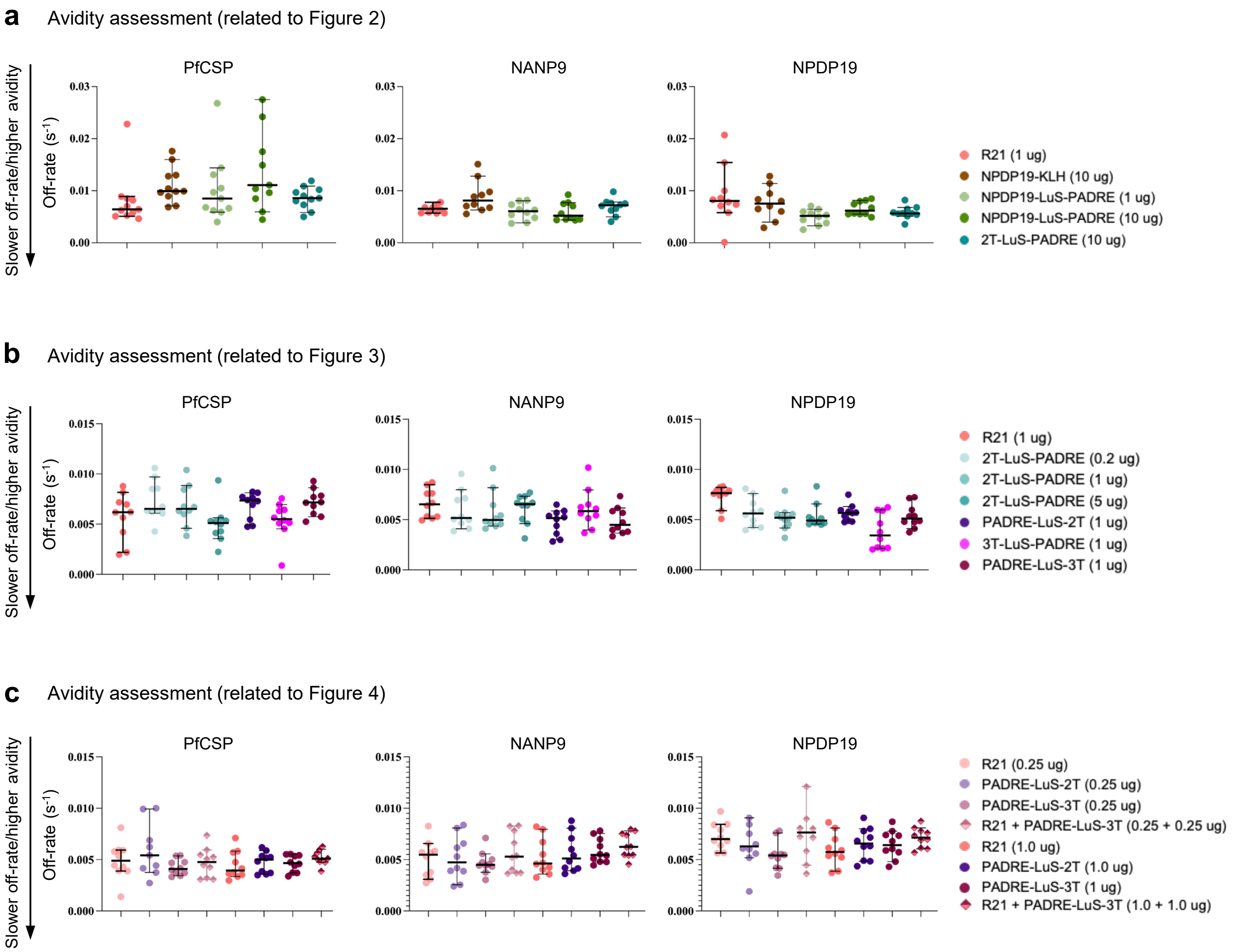

Supplementary Figure 3. Serum antibody avidity analyses, related to Fig 2, 3, 4.
The off-rate of polyclonal sera was measured using biolayer interferometry (BLI). Off-rate constants were determined by fitting the dissociation phase to a 1:1 binding model. The left panel shows avidity to PfCSP, the middle panel shows avidity to the major repeat region (NANP9), and the right panel shows avidity to the junction region (NPDP19).

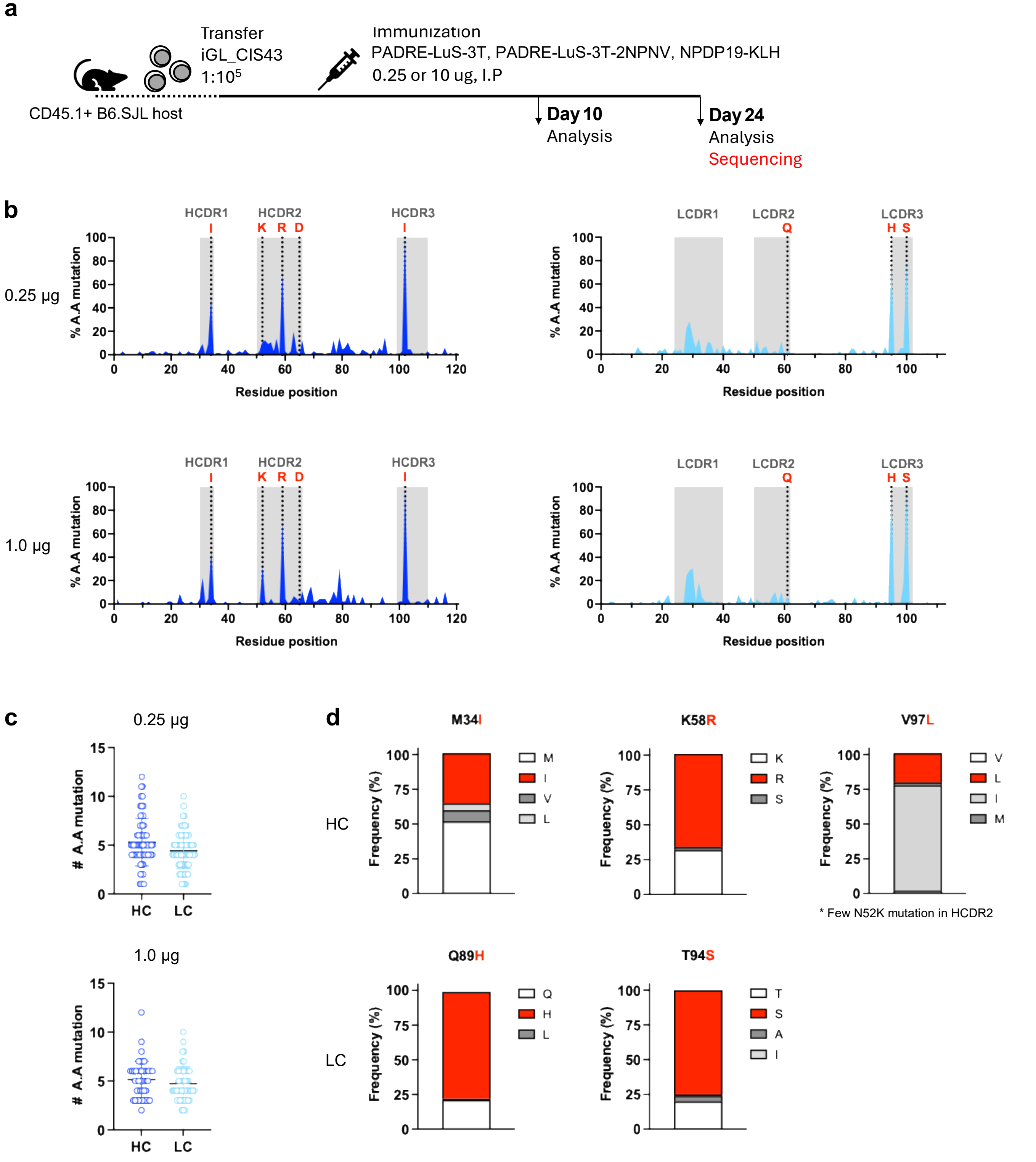

Supplementary Figure 4. Mutational frequency from iGL-CIS43 mice immunized with PADRE-LuS-3T, related to Fig. 5.

a Schematic of adoptive transfer model. **b** Hotspot analysis shows frequency of observed HC and LC mutations per residue at 24 DPI. HCDRs and LCDRs are highlighted in gray. Letters in red (only present in mature CIS43 HC) indicate key aa residues for the recognition of the junctional epitope. **c** Total amino acid mutations for HC and LC are shown. **d** Distribution frequency of select iGL-CIS43 B cell HC and LC aa mutations at positions 34, 58, 97, 89, and 94.

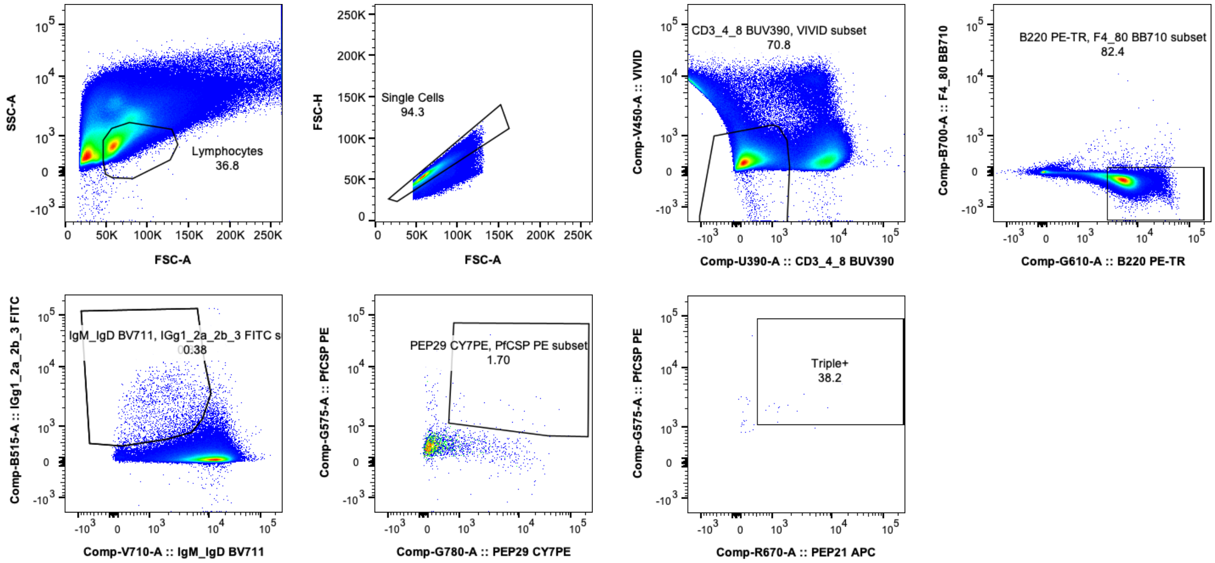

Supplementary Figure 5. B-cell sorting data, related to Fig. 6.
B-cell sorting steps to collect PfCSP/P21/P29 triple positive IgG+ B cells for RATPIg. P21 (junction); P29 (major repeat).
